# Impact of a cafeteria diet and daily physical training on the rat serum metabolome

**DOI:** 10.1371/journal.pone.0171970

**Published:** 2017-02-13

**Authors:** Susana Suárez-García, Josep M. del Bas, Antoni Caimari, Rosa M. Escorihuela, Lluís Arola, Manuel Suárez

**Affiliations:** 1 Departament de Bioquímica i Biotecnologia, Nutrigenomics Research Group, Universitat Rovira i Virgili, Tarragona, Spain; 2 Technological Unit of Nutrition and Health, EURECAT-Technological Center of Catalonia, Reus, Spain; 3 Institut de Neurociències, Departament de Psiquiatria i Medicina Legal, Universitat Autònoma de Barcelona, Barcelona, Spain; Universidad Pablo de Olavide, SPAIN

## Abstract

Regular physical activity and healthy dietary patterns are commonly recommended for the prevention and treatment of metabolic syndrome (MetS), which is diagnosed at an alarmingly increasing rate, especially among adolescents. Nevertheless, little is known regarding the relevance of physical exercise on the modulation of the metabolome in healthy people and those with MetS. We have previously shown that treadmill exercise ameliorated different symptoms of MetS. The aim of this study was to investigate the impact of a MetS-inducing diet and different intensities of aerobic training on the overall serum metabolome of adolescent rats. For 8 weeks, young rats were fed either standard chow (ST) or cafeteria diet (CAF) and were subjected to a daily program of training on a treadmill at different speeds. Non-targeted metabolomics was used to identify changes in circulating metabolites, and a combination of multivariate analysis techniques was implemented to achieve a holistic understanding of the metabolome. Among all the identified circulating metabolites influenced by CAF, lysophosphatidylcholines were the most represented family. Serum sphingolipids, bile acids, acylcarnitines, unsaturated fatty acids and vitamin E and A derivatives also changed significantly in CAF-fed rats. These findings suggest that an enduring systemic inflammatory state is induced by CAF. The impact of physical training on the metabolome was less striking than the impact of diet and mainly altered circulating bile acids and glycerophospholipids. Furthermore, the serum levels of monocyte chemoattractant protein-1 were increased in CAF-fed rats, and C-reactive protein was decreased in trained groups. The leptin/adiponectin ratio, a useful marker of MetS, was increased in CAF groups, but decreased in proportion to training intensity. Multivariate analysis revealed that ST-fed animals were more susceptible to exercise-induced changes in metabolites than animals with MetS, in which moderate-intensity seems more effective than high-intensity training. Our results indicate that CAF has a strong negative impact on the metabolome of animals that is difficult to reverse by daily exercise.

## Introduction

Metabolic syndrome (MetS) is a combination of metabolic disturbances, including insulin resistance, dyslipidemia, obesity and hypertension, that is becoming increasingly prevalent in our society due to sedentary lifestyles and dietary patterns [1–3]. Considering that this disorder may result in cardiovascular disease [4] and type II diabetes [5], great efforts are being made to prevent its development. It has been shown that physical activity provides beneficial effects, including weight loss, changes in body fat percentage, and improvements in blood pressure, lipoprotein profile, cholesterol levels and insulin sensitivity [6–8]. Particularly in adolescents, the substitution of sedentary habits such as watching television for many hours with increased physical activity is the main strategy for the prevention and treatment of obesity [9].

A good model for studying MetS is the cafeteria diet (CAF)-fed rodent, as it develops all the complications present in the human syndrome [10,11]. The CAF-fed rat model is a robust model for diet-induced human obesity associated with muscle, adipose tissue and serum inflammation that leads to the development of MetS [12]. Cigarroa *et al*. [13] showed that CAF administered to weaning rats for 8 weeks induced a sharp rise in body and relative retroperitoneal white adipose tissue (% RWAT) weight, showing these animals a significant increase of 30% and 120% on these parameters, respectively, when compared with rats fed a standard chow (ST). Furthermore, CAF intake during this period of time also produced hyperleptinemia, hypertriglyceridemia, hyperglycemia and insulin resistance. Although in that study, a treadmill intervention at low (12 m/min) or high (17 m/min) intensity did not produce a body weight-lowering effect in CAF-fed animals, we demonstrated that daily physical training had beneficial effects on RWAT weight and triglycerides, partially counteracting the increase in these parameters induced by CAF. In the present study, we provide an omics approach to investigate, in the same cohort of animals, the impact of the chronic intake of CAF and different intensities of daily exercise on the overall serum metabolome of adolescent rats.

Some researchers have attempted to identify distinctive metabolite profiles associated with obesity in adults [14] and youths [15]. All these studies point to the importance of biomarker discovery for early diagnosis, treatment and assessment of lifestyle-related diseases. Recent studies have applied comprehensive metabolomics to uncover metabolite perturbations in CAF-fed rats [12,16] and in trained rodents [17,18]; however the combined influence of CAF and daily physical activity on the circulating metabolome has not yet been investigated.

The objectives of this study were (1) to identify novel circulating metabolites influenced by chronic CAF intake in rats, (2) to elucidate further changes in the metabolome that may help us understand the mechanisms whereby periodic training exerts its beneficial (or detrimental) effects in young animals, (3) to examine whether differences exist between the effects of low- and high-intensity exercises, and (4) to verify whether these biochemical processes differ between animals with and without MetS.

Our goal was accomplished using liquid chromatography coupled to mass spectrometry (LC-MS/MS), a powerful technique very suitable for metabolomics studies that allows the identification of molecular alterations among experimental groups. The use of comparative untargeted metabolomics allows for the identification of potential biomarkers and characteristic metabolic signatures that may be predictive of the health status of the animals.

## Material and methods

### Chemicals

Acetonitrile (Merck, Darmstadt, Germany), glacial acetic acid (Panreac, Barcelona, Spain), methanol and formic acid (Scharlab S.L., Barcelona, Spain) were of high-performance liquid chromatography (HPLC) analytical grade. Ultrapure water was obtained using a Milli-Q advantage A10 system (Madrid, Spain). For mass spectrometry, paracetamol and pyrocatechol (Fluka/Sigma-Aldrich, Madrid, Spain) were used as internal standards in positive (+ESI) and negative (-ESI) electrospray ionization mode, respectively. Both were dissolved in methanol at 1 mg/mL and stored at -20°C.

### Animals, diets and training sessions

Female Sprague-Dawley rats weighting 62 ± 2 g were weaned at 21–23 days of age and housed 2 per cage at 22°C with a light/dark period of 12 h. Female animals were selected because they tolerate higher intensities of forced exercise compared to males and are more active in the voluntary wheel running [19,20]. The animals were randomly distributed into 6 groups (n = 9–12) according to the diet (ST or CAF) and the intensity of the treadmill intervention received during 8 weeks: control-ST (CON-ST), treadmill-low intensity-ST (TML-ST), treadmill-high intensity-ST (TMH-ST), control-CAF (CON-CAF), treadmill-low intensity-CAF (TML-CAF) or treadmill-high intensity-CAF (TMH-CAF).

The CAF included the following components (quantity per rat/day): ST (6–10 g), bacon (8–12 g), biscuits with pâté (12–15 g) or cream cheese (10–12 g), sweet roll (8–10 g), carrot (6–9 g) and milk with sugar (220 g/L; 50 mL). ST had a calorie breakdown of 24% protein, 18% fat and 58% carbohydrates, whereas CAF had 10% protein, 41% fat and 49% carbohydrates [21]. The animals consumed tap water and diet *ad libitum* throughout the experiment, and CAF was renewed daily.

Training sessions were performed as previously described [13]. Briefly, the rats were trained on a treadmill 5 days per week for 30 min. Initially, the animals were accustomed to the treadmill (0 m/min), and the speed was progressively increased until it reached 12 m/min in TML and 17 m/min in TMH groups. These values were maintained until the end of the experiment. Neither electrical shock nor physical prodding was used to motivate the animals. The control rats stayed on the treadmill (0 m/min) for an equivalent amount of time as the trained rats.

The animals were fasted for 12 h and then sacrificed by beheading to avoid interferences in the circulating metabolome due to drugs. Total blood was collected and serum was obtained by centrifugation at 2,000 g for 15 min after 1 h at room temperature.

### Ethics statement

All procedures were approved by the Generalitat de Catalunya (DAAM 6836) and they have been performed in accordance with the European Communities Council Directive (86/609/EEC).

### Determination of serum lipids, hormones and cytokines

Enzymatic kits were used to determine triglycerides, total cholesterol (QCA, Barcelona, Spain) and high- and low-density lipoprotein cholesterols (HDLc and LDLc; Spinreact, Girona, Spain). Leptin, adiponectin, C-reactive protein (CRP) (Millipore, Barcelona, Spain) and monocyte chemoattractant protein-1 (MCP-1) (Thermo Fisher Scientific, Pittsburgh, USA) were measured using rat ELISA kits. The irisin levels were determined using a human/rat/mouse ELISA kit (Phoenix Pharmaceuticals Inc., Burlingame, California, USA).

### Sample preparation for metabolomics analysis

Metabolites were extracted from serum using a hydroalcoholic solution. 800 μL of methanol:water (8:1 vol/vol) were added to 100 μL of serum, and 50 μL of pyrocatechol (1 ppm) and 50 μL of paracetamol (1 ppm) were added as internal standards. The mixture was homogenized by vortexing (30 s) and ultrasonication (30 s). Then, samples were incubated on ice for 10 min to precipitate proteins and centrifuged at 19,500 g for 10 min at 4°C. The supernatant was collected and dried under nitrogen flow to eliminate the solvent. Finally, dried samples were re-dissolved in 200 μL of methanol:water (8:1 vol/vol) prior to injection.

### LC-MS and LC-MS/MS analysis

Non-targeted analysis of the serum extracts was performed using an HPLC 1200 series coupled to an ESI-TOF 6210 (Agilent Technologies, Palo Alto, California, USA). Each sample (7.5 μL) was injected into a Zorbax SB-Aq (3.5 μm particle size, 2.1 mm internal diameter x 150 mm length) chromatographic column equipped with a Zorbax SB-C18 (3.5 μm, 2.1 x 15 mm) pre-column, also from Agilent Technologies. The ionization in the mass spectrometer was performed in +ESI and -ESI to cover all the ranges of metabolites. In +ESI, the mobile phase consisted of 0.1% formic acid (solvent A) and acetonitrile with 0.1% formic acid (solvent B). In -ESI, solvent A was 0.2% acetic acid, and solvent B was acetonitrile. The chromatographic separation was performed using a continuous gradient elution at flow rate of 0.4 mL/min starting at 10% B and increasing to 100% B over 45 min, after which time it was maintained for 10 min. The ionization source parameters were as follows: nitrogen was used as the nebulizer gas with a pressure of 45 psi; desolvation gas flow rate, 9 L/min at 325°C; source temperature and gas flow rate, 150°C and 12 L/min, respectively; capillary voltage 4 kV; and fragmentor was set to 125 V. LC-MS accurate mass spectra were acquired from 50 to 1200 *m/z* at a scan rate of 1 spectra/s. A reference solution was used for the continuous calibration using the following reference masses: 121.0509 and 922.0098 *m/z* for +ESI and 119.0363 and 980.0164 *m/z* for -ESI.

For targeted analyses, extracts were injected in a UHPLC 1290 series coupled to a Q-TOF 6550, also from Agilent Technologies, operated either in MS or MS/MS modes. The LC-MS/MS analyses were performed using the same analytical conditions described above. The data were collected in the range of *m/z* 100–1000 with a scan rate of 1.5 spectra/s. The MS/MS spectra of metabolites were obtained by different collision energies (10, 20 and 40 eV).

### Untargeted data processing and metabolite identification

All the software programs used to process the data were also from Agilent Technologies. Untargeted data were acquired using MassHunter Data Acquisition, whereas Qualitative Analysis was used to obtain the molecular features of the samples using the ‘Molecular Feature Extractor’ algorithm, which removes the background ions and groups related ions in a unique feature based on the presence of adducts and dimers, the isotopic distribution and the charge-state envelope. Mass Profiler Professional was used to perform the alignment of the features present in the chromatograms and to carry out multivariate statistical analysis. Data from each ionization mode, positive and negative, were analysed separately. A list of chemical entities was obtained, and only those features with a minimum of 2 related ions were selected for the subsequent chemometric analysis. Multiple charge states were not considered. The retention time and mass window used for alignment were 0.5% ± 0.15 min and 10.0 ppm ± 2.0 mDa, respectively. Abundance values corresponding to each entity were transformed to a base-2 logarithm and normalized to the internal standard. Uncommon features were discarded, and only those that were found in at least 75% of the samples within the same group were selected.

A multivariate analysis based on a combination of hierarchical clustering analysis (HCA), principal components analysis (PCA), and partial least squares for discriminant analysis (PLS-DA) was performed to evaluate the influence of diet and physical exercise on the metabolome. The HCA was conducted on both features and conditions. The distance metric selected for the analysis was euclidean, and the linkage rule was centroid. Analysis of variance (ANOVA) was used to detect significant differences in the normalized abundances of the filtered features. To handle false discovery rates from multiple comparisons, the cut-off point for significance was calculated according to the Benjamini-Hochberg correction [22] at a level of 5%. All significant masses were extracted manually from the chromatograms to verify that they were in sufficient abundance in the spectra at the specific retention time to be subsequently fragmented by LC-MS/MS. After cleaning the spectrum for each entity, the molecular formula generator algorithm included in the MassHunter Qual software was used to obtain a list of candidate molecular formulas. This algorithm considers more information contained in the spectra than the value of the monoisotopic mass (isotope abundance, spacing between isotope peaks, etc.) [23]. Taking into consideration all the information about the nature of the entities, various databases such as METLIN [24], HMDB [25], LIPID MAPS [26] and KEGG [27] were used for the tentative identification of the molecules. Finally, to confirm the identity of the metabolites of interest, LC-MS/MS was used to obtain the fragmentation patterns of the molecules and these were compared with spectral information for the candidates.

### Univariate statistical analysis

After this previous screening, several metabolites were identified and studied more thoroughly. The differences among groups were assessed using one- and two-way ANOVA. First, two-way ANOVA was carried out to evaluate the effects of diet, exercise and their interactions. The results were reported in tables and bar charts with italic capital letters indicating a significant effect of diet (*D*), exercise (*E*) or their interaction (*DxE*). When one or both main effects were statistically significant, one-way ANOVA was used to determine the differences between the means. When only the interaction between diet and exercise was statistically significant according to the two-way ANOVA model, one-way ANOVA was carried out to compute pairwise comparisons between diet groups (i.e., the effect of exercise within diet groups). The assumption of normality was determined using the Shapiro-Wilk test, and the homoscedasticity between groups was determined using Levene’s test. Tukey *post hoc* contrast was used when variances between groups were similar, and Dunnett’s T3 test was used if this assumption was not fulfilled. Data are expressed as the means with their standard errors (SEM), and the results of the *post hoc* contrasts are shown using lower-case letters. Student’s t-test was used for single statistical comparisons. A two-tailed value of p<0.05 was considered statistically significant for all tests. Statistical analyses were performed using the Statistical Package for Social Sciences (IBM SPSS Statistics, ver19.0).

## Results

### Targeted analysis of serum metabolites

#### Dyslipidemic parameters

**Fig 1:**
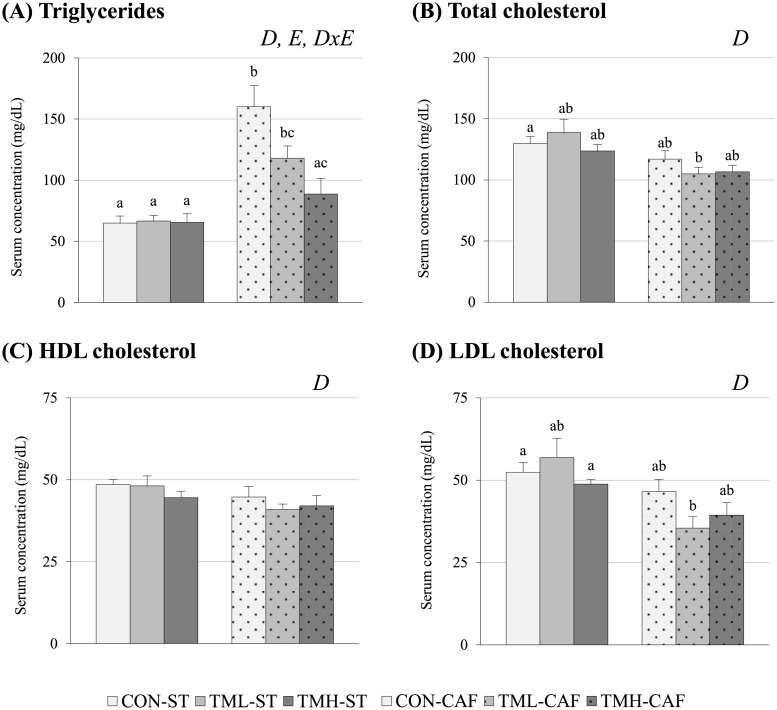
Dyslipidemic parameters. Animals were distributed into 6 groups (n = 9–12) based on diet and training condition: control-standard diet (CON-ST), treadmill-low intensity-standard diet (TML-ST), treadmill-high intensity-standard diet (TMH-ST), control-cafeteria diet (CON-CAF), treadmill-low intensity-cafeteria diet (TML-CAF) and treadmill-high intensity-cafeteria diet (TMH-CAF). Diets and training sessions started after the weaning period and were extended for 8 weeks. Lipid serum concentrations were determined at the end of the experiment after 12 h of fasting. The data are given as the mean ± SEM. The statistical comparison among groups was conducted using two- and one-way ANOVA. *D*: the effect of diet; *E*: the effect of exercise; *DxE*: the interaction between the two main factors. ^abc^Mean values with different small letters indicate significant differences between groups (one-way ANOVA and Dunnett’s T3 *post hoc* contrast, p<0.05).

We have previously shown in Cigarroa *et al*. [13] that CAF-fed rats have higher amounts of triglycerides in their serum than animals fed ST, and this increase gradually drops with exercise until triglycerides nearly return to ST-fed levels in the TMH-CAF group (Fig 1A). CAF feeding also had significant effects on the remaining dyslipidemic variables (p<0.05, two-way ANOVA). Curiously, CAF-fed animals had lower circulating levels of total cholesterol, HDLc and LDLc than ST-fed groups. (Fig 1B and 1D). Interestingly, although LDLc levels remained unchanged among ST-fed animals, a significant decrease was detected in the serum of CAF-fed rats as a result of running at low-intensity compared to the sedentary group (p = 0.04, Student’s t-test) (Fig 1D).

#### Leptin/adiponectin ratio (LAR)

**Fig 2:**
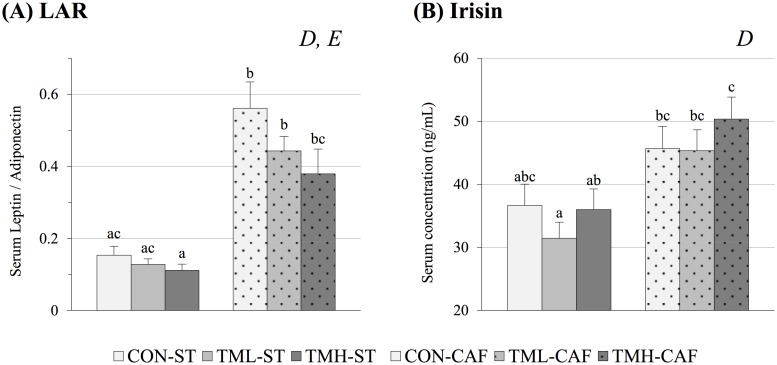
Circulating levels of hormones associated with lifestyle-related diseases. Animals were fed standard chow (ST) or cafeteria diet (CAF) for 2 months and periodically trained on a treadmill at different intensities (CON: 0; TML: 12; TMH: 17 m/min). The serum levels of adiponectin, leptin and irisin were determined at the end of the experiment after 12 h of fasting. The data are given as the mean ± SEM (n = 9–12). The statistical comparison among groups was conducted using two- and one-way ANOVA. *D*: the effect of diet; *E*: the effect of exercise. ^abc^Mean values with different small letters were significantly different (one-way ANOVA and Tukey/ Dunnett’s T3 *post hoc* contrast, p<0.05).

We observed a clear effect of both CAF feeding (p<0.01) and physical exercise (p<0.05, two-way ANOVA) on LAR (Fig 2A). This index was higher in CAF-fed rats than in ST-fed animals, and this increase was progressively reversed by the practice of running until it practically returned to ST-fed levels in the animals trained at high-intensity. Moreover, LAR was the only parameter on which physical exercise had significant effects in ST-fed groups. The progressive reduction of the ratio due to training was observed in animals regardless of diet.

#### Circulating levels of the exercise hormone irisin

Contrary to what was expected, the levels of irisin were largely affected by diet (p<0.01, two-way ANOVA) and not by physical training. As shown in Fig 2B, CAF feeding induced elevated circulating levels of this hormone, especially in the group of high-intensity runners.

#### Systemic inflammatory levels

**Fig 3:**
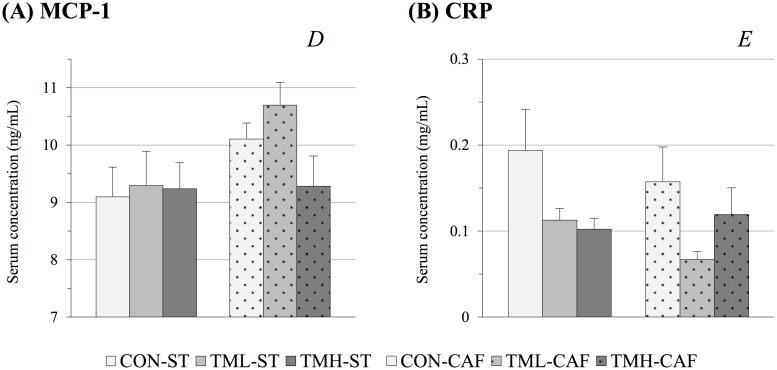
Circulating inflammatory markers. Animals were fed standard chow (ST) or cafeteria diet (CAF) and trained 5 days per week for 30 min on a treadmill at different intensities (CON: 0; TML: 12; TMH: 17 m/min). Diets and training sessions were continued for 8 weeks. The serum levels of the cytokines monocyte chemoattractant protein-1 (MCP-1) and C-reactive protein (CRP) were determined at the end of the experiment. The data are given as the mean ± SEM (n = 9–12). *D*: the effect of diet; *E*: the effect of exercise (p<0.05, two-way ANOVA).

Due to the pro-inflammatory effects of CAF consumption in rodents, the serum concentrations of two inflammatory markers were assessed (Fig 3). The CAF-fed animals had increased levels of the chemotactic cytokine MCP-1 (p = 0.04, two-way ANOVA). Although no significant effect of physical training on MCP-1 was established by two-way ANOVA, CAF-fed animals showed a significant decrease due to the treadmill intervention at high-intensity relative to moderate training (p = 0.04, Student’s t-test) (Fig 3A). Contrasting MCP-1, serum levels of the cytokine CRP were influenced only by physical exercise (p = 0.04, two-way ANOVA). In ST-fed rats, the exercise produced a similar decline (approximately 45%) in CRP levels in both trained groups relative to those of the CON-ST animals. Remarkably, CRP values of CAF-fed rats experienced the highest decrease (of 60%) relative to the sedentary group as a consequence of moderate training (p<0.05, Student’s t-test), whereas TMH-CAF levels were similar to those for the CON-CAF group (Fig 3B). These findings suggest that the two training intensities did not yield similar effects on inflammation levels in CAF-fed animals.

### Untargeted evaluation of circulating metabolome

Analysis of the LC-ESI-MS data revealed that 6,594 molecular features were aligned in +ESI. From these, 1,207 features were present in at least 75% of the samples within the same group. These features were selected for subsequent analysis. Similarly, when analysing data from -ESI 5,973 molecular features were aligned. Of these, the 1,479 features present in at least 75% of the samples within the same group were selected.

**Fig 4:**
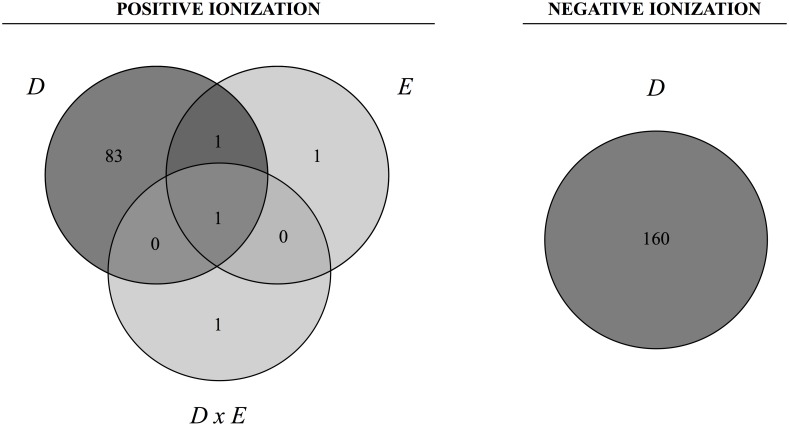
Venn diagrams showing the number of significant entities from each experimental parameter. Data from each ionization mode, positive and negative, were analysed using two-way ANOVA. To handle false discovery rates from multiple comparisons, the cut-off point for significance was calculated according to the Benjamini-Hochberg correction at a level of 5%. *D*: the effect of diet; *E*: the effect of exercise; *DxE*: the interaction between the two main factors. The areas where the circles overlap show the number of significant entities shared by the parameters.

By means of two-way ANOVA adjusted using the Benjamini-Hochberg correction, we found 87 and 160 metabolites with altered concentration between groups (p(Corr)<0.05) in +ESI and -ESI, respectively. As shown in the Venn diagram (Fig 4), these significant differences were mainly due to diet rather than physical exercise in +ESI. However, in -ESI, there were no significant differences observed due to exercise. The complete results of the non-targeted analysis are provided in S1 Table.

**Fig 5:**
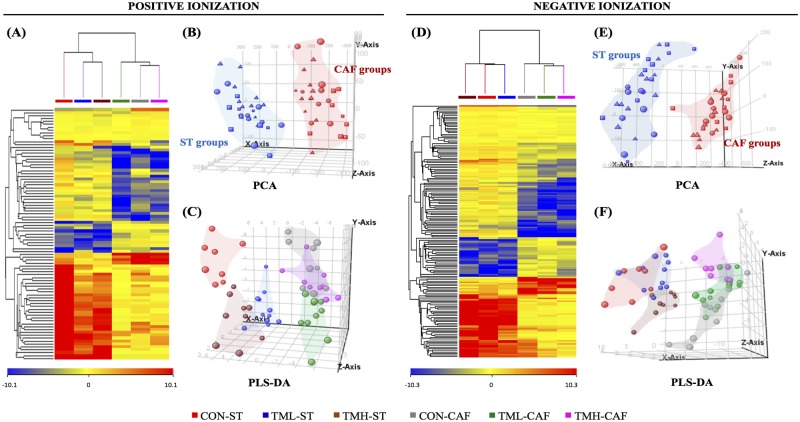
Multivariate analysis demonstrating the effect of diet and physical exercise on the animal metabolome. Serum extracts were analysed using LC-ESI-MS in both positive and negative ionization modes. (A, D) Heat map representations of hierarchical clustering of significant entities found in each group of animals. Each row represents an exact mass coloured by its abundance intensity, normalized to an internal standard and baselined to the mean of all samples. The scale from -10 (blue) to +10 (red) represents this normalized abundance in arbitrary units. The PCA (B, E) and PLS-DA (C, F) graphs show that the effect of cafeteria diet had prevalence over the periodic training on the treadmill at diverse intensities. Abbreviations: ST, standard chow; CAF, cafeteria diet; CON, control animals; TML, treadmill-low intensity runners; TMH, treadmill-high intensity runners.

To evaluate the influence of daily exercise and chronic intake of CAF on the metabolome, we used a combined multivariate analysis. The relative average normalized abundances of the differential metabolites were plotted using a heat map (Fig 5A and 5D), and in both ionization modes, the importance of the two parameters was evident, although the influence of diet was far greater than those exerted by training. HCA from +ESI data also indicated that the effect of exercise was more marked when animals were fed ST rather than CAF. Interestingly, while both exercise intensities caused a similar change in the metabolome of ST-fed groups, when animals were fed CAF, the practice of running at a low speed influenced the metabolome in a diverse manner compared to what happened in the other two groups of CAF-fed rats, approaching the TML-CAF metabolome to ST-fed rats (Fig 5A). PCA of +ESI and -ESI data, explaining a 43% and 47% of the variance, respectively, resulted in a clear clustering of animals depending on diet but not on physical activity (Fig 5B and 5E). PLS-DA was used to assess whether the 6 groups could be clustered separately using a supervised approach (Fig 5C and 5F). The classification accuracies associated to the predictive PLS-DA model were 82% and 85% in +ESI and -ESI, respectively, suggesting an acceptable performance and therefore a good clustering trend.

#### Metabolites associated with cafeteria diet consumption

Having determined the entities that were significantly modified by diet, a tentative identification of their nature was carried out. This identification was performed by LC-ESI-MS/MS using a comparison of exact mass, retention time, and spectral and fragmentation information with those in metabolite databases (Table 1). The results indicate that CAF altered the levels of seven of the eight lipid categories included in LIPID MAPS: glycerophospholipids, mainly lysophosphatidylcholines (Lyso-PC); sphingolipids and glycerolipids, both increased in CAF-fed animals; sterols such as bile acids, prenol lipids including vitamin E derivatives and retinoids and a flavonoid named equol that belongs to the polyketides category, all decreased after chronic intake of CAF; and a fatty acyl category consisting of unsaturated free fatty acids and acylcarnitines, which decreased and increased, respectively, in the serum of CAF-fed animals. Lyso-PC was the most represented family influenced by diet with a heterogeneous regulation in response to CAF feeding.

**Table 1:** Identified metabolites that differed significantly with the diet.

Category	Compound	Entity (M@RT) ^╪^	Fragmentation pattern *	Collision Energy (V)	Formula	Abundance in CAF groups
**Glycerophospholipids**	Lyso-PC (16:1)	493.3171@22.3	184.1, 104.1, 86.1	40	C_24_H_48_NO_7_P	Increased
	Lyso-PC (17:1)	507.3325@23.5	184.1, 104.1, 86.1	40	C_25_H_50_NO_7_P	
	Lyso-PC (18:0)	523.2944@24.1	184.1, 104.1, 86.1	40	C_26_H_54_NO_7_P	
	PAF	637.3937@27.5	184.1, 86.1	20	-	
	Lyso-PE (O-18:0)	467.3378@25.4	447.3, 429.3	20	C_23_H_50_NO_6_P	Decreased
	Lyso-PC (20:2)	547.364@25.7	184.1, 104.1, 86.1	40	C_28_H_54_NO_7_P	
	Lyso-PC (20:1)	549.3788@27.2	184.1, 104.1, 86.1	40	C_28_H_56_NO_7_P	
**Sphingolipids**	Sphinganine	301.2975@21.7	60.0, 284.3, 254.3	20	C_18_H_39_NO_2_	Increased
	Sphinganine-1-phosphate	381.2639@21.3	266.3, 284.3	20	C_18_H_40_NO_5_P	
**Glycerolipids**	MG (16:0)	330.2759@30.1	57.1, 71.1, 85.1	20	C_19_H_38_O_4_	Increased
**Sterol lipids**	Glycocholic acid	465.3089@17.2	76.0, 209.1, 319.2, 337.2, 412.3	40	C_26_H_43_NO_6_	Decreased
	Hydroxylated bile acid	406.2714@18.2	145.1, 107.1, 197.1, 81.1	40	C_24_H_38_O_5_	
	Hydroxylated bile acid	390.2775@22.2	145.1, 109.1, 57.1, 199.1	40	C_24_H_38_O_4_	
**Free fatty acids: PUFA**	Linolenic acid (18:3)	278.2242@30.2	81.1, 95.1, 67.1, 121.1	20	C_18_H_30_O_2_	Decreased
	DHA (22:6)	328.2391@31.7	91.1, 79.1, 67.1, 55.1	40	C_22_H_32_O_2_	
**Free fatty acids: MUFA**	DiHOME (18:1)	314.2465@22.2	67.1, 81.1, 279.2	20	C_18_H_34_O_4_	Decreased
**Fatty acid esters: Carnitines**	Butyryl carnitine (C4)	231.1471@1.99	85.0, 57.0	40	C_11_H_21_NO_4_	Increased
	Myristoyl carnitine (C14)	371.3033@22.3	85.0, 60.1	40	C_21_H_41_NO_4_	
**Prenol lipids**	γ-CEHC	264.1365@16.1	151.1, 123.1, 95.1, 67.1	40	C_15_H_20_O_4_	Decreased
	α-CEHC	278.1514@17.6	165.1, 137.1, 122.1, 67.1	40	C_16_H_22_O_4_	
	Retinoyl glucuronide	476.2396@17.7	255.2, 301.2	20	C_26_H_36_O_8_	
**Polyketides: Flavonoids**	Equol	242.0953@11.4	123.0, 133.0, 107.0	10	C_15_H_14_O_3_	Decreased
**Other benzenoids**	Phenyl butyrate	164.0834@17.6	77.0	40	C_10_H_12_O_2_	Decreased
	Hippuric acid	179.059@4.49	77.0, 51.0, 105.0	40	C_9_H_9_NO_3_	

^╪^ Each entity comprises a particular neutral mass (M) and retention time (RT), and both values are separated by the symbol “@”

* Distinctive fragments from LC-+ESI-MS/MS analysis at the most appropriate collision energy for each molecule

Abbreviations: CAF, cafeteria diet; Lyso-PC, lysophosphatidylcholine; PAF, platelet activating factor; Lyso-PE, lysophosphatidylethanolamine; MG, monoacylglycerol; PUFA, polyunsaturated fatty acid; DHA, docosahexaenoic acid; MUFA, monounsaturated fatty acid; DiHOME, dihydroxy-octadecenoic acid; γ-CEHC, gamma-tocopheronolactone; α-CEHC, alpha-tocopheronolactone.

#### Changes in metabolome related to physical activity

**Fig 6:**
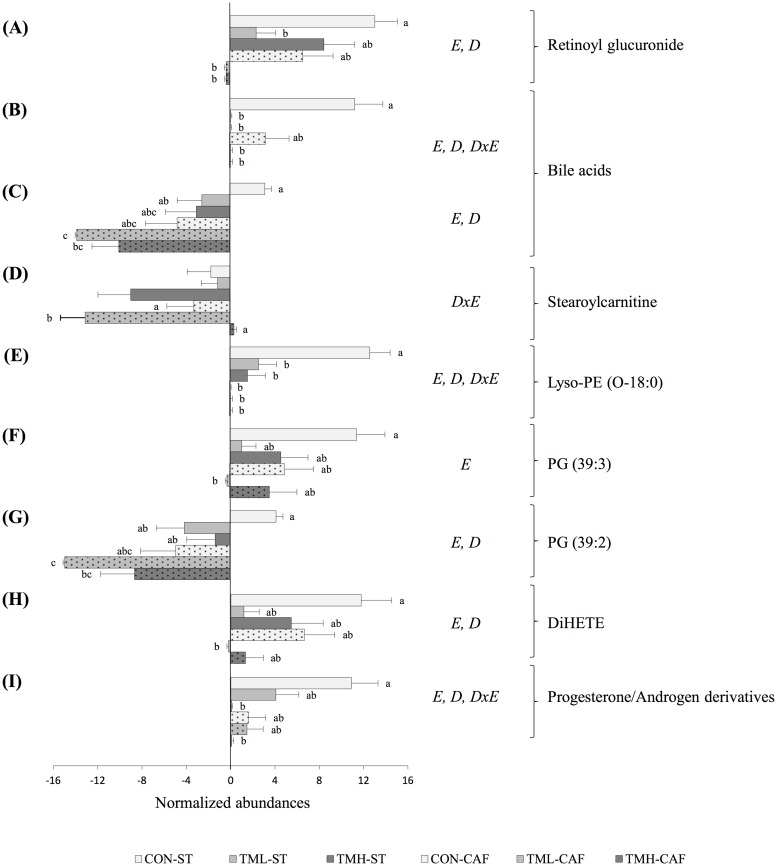
Identified metabolites altered with the periodic practice of running on a treadmill at diverse intensities. The abundance values for each metabolite were transformed to a base-2 logarithm, normalized by the internal standard, and baselined to the mean of all samples. The data are given as the mean ± SEM (n = 9–12). To avoid the occurrence of false positives, a primary screen was carried out using two-way ANOVA and adjusting the p-value of the exercise parameter using the Benjamini-Hochberg correction. To more exhaustively evaluate the differences among groups, a posterior analysis without a p-value correction was performed. *E*: the effect of exercise; *D*: the effect of diet; *DxE*: the interaction between the two factors (two-way ANOVA, p<0.05). ^abc^Mean normalized values with different small letters were significantly different (one-way ANOVA and Tukey/Dunnett’s T3 *post hoc* contrast, p<0.05). Abbreviations: Lyso-PE, lysophosphatidylethanolamine; PG, phosphatidylglycerol; DiHETE, dihydroxy-eicosatetraenoic acid.

Focusing on the metabolites on which physical exercise had a significant effect, four biomarkers (Fig 6A, 6B, 6D and 6E) were identified. Of these, three were directly modified by exercise (retinoyl glucuronide, a lysophosphatidylethanolamine, and a bile acid), as their serum concentrations significantly decreased with the practice of running in both ST and CAF-fed rats. A significant interaction between diet and exercise was found for the levels of stearoylcarnitine, which were significantly lower in TML-CAF rats compared to those of the CON-CAF and the TMH-CAF groups (Fig 6D).

Furthermore, the HCA of CAF-fed groups indicates that there is a differential effect on the metabolome between the two groups of rats submitted to different intensities of exercise. Contrary to what was expected, the more intense training approaches the metabolome of TMH-CAF rats to the CON-CAF group. This may suggest that the level of exercise influences the metabolome in a different manner, and essential information, such as exercise biomarkers, may be masked and lost. Therefore, we continued the restrictive statistical analysis comparing the CON conditions with each training level separately. Six additional exercise biomarkers were discovered, from which five were effectively identified (Fig 6C and 6F–6I). Again, all metabolites were lipids, namely a steroid hormone derivative, the dihydroxy-octadecenoic acid known as DiHETE, two phosphatidylglycerols, and a sexual hormone derivative. All of these were down-regulated by exercise: the bile acid, both phosphatidylglycerols and DiHETE were reduced by low-intensity running, and the unidentified sexual hormone metabolite was reduced by high-intensity running.

## Discussion

This study is the first to apply a non-targeted metabolomics approach to investigate the impact of the combination of CAF and regular exercise training on the circulating metabolome. All of the animals within each group responded consistently to the treatments, ranging the variance from 1.9 to 4.7% RSD in relation to body weight gain [13]. This suggests that all the differences observed in the metabolome are due to the treatments evaluated, diet and exercise. Thus, we have shown that CAF feeding administered from a weaning age and continued until late adolescence leads to the accumulation of acylcarnitines in the serum of rats (Table 1), suggesting a disruption in fatty acid oxidation. This is in accordance with Sampey *et al*. [12], who showed that this increase is also appreciable in muscle and white adipose tissue of CAF-fed rats, suggesting that the results found in the blood reflects the tissue’s acylcarnitine metabolism. Human studies have also revealed the implications of acylcarnitines in youth with obesity and diabetes [15]. Interestingly, herein, the practice of running at low-intensity had an opposite effect if we consider the decreased circulating abundance of stearoylcarnitine in low-intensity runners fed CAF (Fig 6D). Our results suggest that the chronic exposure of muscle to elevated lipids resulted in the over-expression of genes involved in fatty acid β-oxidation leading to the accumulation of acylcarnitines, and exercise might ameliorate mitochondrial activity and counteract this over-expression [28].

We found two sphingolipids, sphinganine and sphinganine-1-phosphate, whose levels were elevated in CAF-fed animals (Table 1). Sphingolipids are synthetized in hepatocytes in a process that involves inflammatory cytokines and are then incorporated into lipoproteins. The alteration of the lipoprotein composition may be responsible for the increased atherogenicity of these particles [29,30]. Relatedly, previous studies have shown that sphingolipids contribute not only to atherosclerosis but also to insulin resistance, diabetes and cancer [31–33]. Specifically, plasma sphingolipids have been identified as biomarkers of MetS in non-human primates by Brozinick *et al*. [34]. Furthermore, sphinganine-1-phosphate is also released by erythrocytes, and this release is accompanied by an increase in sphingosine, a strong extracellular messenger involved in important cellular functions such as cell migration, proliferation and death [35].

We also detected changes in markers of vitamin A and E consumption and metabolism as a consequence of both CAF and treadmill interventions. The reduction in the serum concentration of retinoyl glucuronide as a consequence of CAF intake (Table 1) can be a signal of vitamin A deficiency. Its role as an exercise biomarker (Fig 6A) may be explained by its capability to serve as reservoir of retinoic acid, which is involved in the regulation of energy homeostasis and insulin responses [36]. On the other hand, α- and γ-tocopheronolactone (α-CEHC and γ-CEHC, respectively) are synthesized from dietary α- and γ-tocopherol, respectively. Many lines of evidence suggest that α-tocopherol has an inhibitory effect on the conversion of arachidonic acid to pro-inflammatory prostaglandins [37,38] and decreases LDLc oxidation [39], pro-inflammatory cytokines and adhesion of monocytes to endothelium [40,41]. Additionally, γ-CEHC and γ-tocopherol molecules exhibited an inhibitory influence on prostaglandin E_2_ synthesis [42,43], a key intermediate in the early inflammatory response with pro-inflammatory activity. The significant decrease observed in tocopherol metabolites as a consequence of CAF intake (Table 1) might be related to the systemic pro-inflammatory and pro-oxidant state of these animals [11,12].

Similarly, we observed decreased bile acids levels in the serum of CAF-fed rats (Table 1), and this decrease was even greater in trained groups (Fig 6B and 6C), which may suggest a disruption in cholesterol homeostasis. This event may also be linked to inflammation because many studies have shown that inflammatory cytokines may inhibit the expression of CYP7A1 and other rate-limiting enzymes in the synthesis of bile acids [44–46].

Long chain omega 3 polyunsaturated fatty acids (ω-3 PUFA) decrease the production of pro-inflammatory eicosanoids from arachidonic acid and support anti-inflammatory eicosanoids production for the purpose of resolving acute inflammation [47,48]. However, in metabolic diseases such as obesity, type II diabetes or atherosclerosis, this situation is unbalanced in favour of pro-inflammatory eicosanoids leading to an enduring inflammatory state [48]. Our data showed decreased levels of ω-3 PUFA (linolenic and docosahexaenoic acid) as a consequence of chronic intake of CAF (Table 1). Therefore, the metabolome of CAF-fed animals reflected nutritional deficiencies that drive disorders in lipid metabolism and inflammation. The serum accumulation of Lyso-PC may be associated with this fact because increased phospholipase A_2_ activity promotes the release of PUFA for subsequent synthesis of bioactive eicosanoids [49]. In fact, Lyso-PC have been linked to acute and chronic inflammation [50,51] as well as obesity [52,53]. Physical training had a subtle effect on PUFA, as only the DiHETE serum concentration was altered. Although increased levels of DiHETE are associated with type II diabetes and other inflammatory diseases [54,55], we observed decreased levels as a consequence of physical exercise, especially at low-intensity (Fig 6H).

Many studies carried out with rodents using a targeted strategy indicate that inflammation is a clear metabolic consequence of chronic CAF consumption [11,56–58]. The results obtained in the present study showed that the pro-inflammatory condition induced by the CAF has a significant holistic effect in the circulating metabolome. In accordance with this, the serum levels of the pro-inflammatory cytokine MCP-1 were increased in CAF-fed rats (Fig 3A). In particular, MCP-1 has been described as the main responsible for the recruitment of monocytes in the inflammatory response, as well as a potential intervention point for the treatment of various diseases associated to MetS and inflammation, including insulin resistance, atherosclerosis and rheumatoid arthritis [59,60]. Some authors have also shown that physical exercise has a significant effect in reducing circulating levels of MCP-1 in rats and subjects with MetS [59,61]. However, our results did not reveal a remarkable influence of the treadmill intervention in serum MCP-1.

Enduring inflammation could lead to a decrease in total and lipoprotein-associated cholesterol [44,62], explaining why we observed the lowest circulating levels of total cholesterol and its fraction associated with lipoproteins in animals fed CAF (Fig 1B–1D). We also observed that the changes in dyslipidemic variables associated with the practice of running were found in animals fed the MetS-inducing diet but not in the ST-fed rodents. Serum triglycerides of CAF-fed animals were clearly modulated by treadmill intervention, decreasing its concentration proportionally to training intensity (Fig 1A). A lowering effect of training is also found in the circulating values of LDLc of TML-CAF rats in comparison with sedentary group (Fig 1D). The lack of modifications in dyslipidemic parameters of animals without MetS may indicate a corrective effect of exercise specifically in dyslipidemia.

Nevertheless, a significant effect of physical training was perceptible in the CRP levels of animals fed both ST and CAF (Fig 3B). In fact, recent studies carried out in obese and non-obese subjects have shown a reduction in the circulating levels of the inflammatory marker CRP in response to regular physical activity [63–65]. In the present study, the low concentrations of CRP found in the serum of trained animals suggest that aerobic training can reduce inflammation. Decreased circulating levels of adiponectin were observed in obese children and adolescents [66,67] and are correlated with MetS and inflammation [2,68]. On the other hand, many studies have concluded that elevated concentrations of plasma leptin are strongly associated with MetS [2,67,69,70]. Because the two adipokines have opposite effects, the plasma LAR has been proposed to be a useful diagnostic marker of MetS in humans [69,70]. According to these authors, we found higher values of LAR in CAF groups relative to the ST-fed rats (Fig 2A). Moreover, the physical training reduced the ratio in both ST and CAF groups, and this response was proportional to training intensity.

We also analysed the serum concentration of the hormone irisin, which has been proposed to mediate some of the beneficial effects of exercise in obesity by driving brown-fat development of the subcutaneous adipose tissue [71]. Surprisingly, we did not detect changes in trained animals, but its levels were increased after CAF intake (Fig 2B). The absence of changes in the irisin levels of trained animals might be related to the fact that it is only transiently expressed after training [72]. To our knowledge, it has never been described that CAF boosts irisin levels; however, recent reports have shown high levels of circulating irisin in obese subjects [73,74]. In fact, at the time of the sacrifice, a higher amount of brown adipose tissue in CAF-fed rats was evident (data not shown). Originally, irisin was identified as a myokine [75], but now it is known that it is also synthetized by white adipose tissue [76]; so it is not surprising that is found in higher concentrations in overweight animals.

Our study used a non-targeted metabolomics approach and suggests that a systemic inflammatory state in adolescent rats is a consequence of the chronic intake of a high-fat and high-carbohydrate diet. The periodic training was only partially able to reverse the detrimental effects induced by the CAF intake, and the moderate exercise seemed more effective than high-intensity training. In fact, multivariate analysis showed that the metabolome of animals fed ST was more affected by the physical training than CAF-fed rats. However, it has to be taken into consideration that the results of the experiment were obtained using female animals. This fact could limit the degree of extrapolation of our results because the metabolome of females is also influenced by the hormonal cycle [77]. Thus it would be necessary to evaluate in subsequent studies if male rats have a similar response regarding overall metabolism when submitted to the same treatments [78].

In future studies, it may also be interesting to analyse, in the same conditions, the hepatic expression of genes related to the metabolism of bile acids, such as CYP7A1 or SREBP2 [79,80], to study the mechanisms through which the chronic inflammation could be hampering the cholesterol homeostasis. Furthermore, it would be appropriate to evaluate the serum concentrations of proteins involved in the extracellular releasing of lysoglycerophospholipids, since they are the family of altered metabolites most representative of our study and are not just linked to inflammatory events but also to other important physiological and pathological processes [81].

From the results of the present study, we conclude that an unhealthy diet has a strong impact on the animal metabolome that is difficult to revert with the practice of running, even when maintained daily.

## Supporting information

S1 Table Complete list of features from the non-targeted metabolomics analysis of the rat serum including their neutral mass, the retention time, abundances and statistical parameters. (XLSX)
